# People with type 2 diabetes’ experiences of emotional support in Australian general practice: a qualitative study

**DOI:** 10.3399/BJGPO.2022.0079

**Published:** 2022-10-19

**Authors:** Rita McMorrow, Barbara Hunter, Nana Folmann Hempler, Kaleswari Somasundaram, Jon Emery, Jo-Anne Manski-Nankervis

**Affiliations:** 1 Department of General Practice, University of Melbourne, Melbourne, Australia; 2 NHMRC CRE in Digital Technology to Transform Chronic Disease Outcomes, The Baker Heart and Diabetes Institute, Melbourne, Australia; 3 Steno Diabetes Centre Copenhagen, Gentofte, Denmark; 4 Centre for Cancer Research, University of Melbourne, Melbourne, Australia

**Keywords:** general practice, diabetes mellitus, type 2, mental health, qualitative study

## Abstract

**Background:**

Diabetes distress, experienced by up to 40% of people with type 2 diabetes (T2D), is the negative emotional response to the burden of living with and managing diabetes. It is associated with suboptimal glycaemia and diabetes self-management. Research indicates that people with diabetes do not recall being asked about emotional distress by healthcare professionals.

**Aim:**

To explore the experiences, needs, and expectations of people with T2D regarding emotional support received in Australian general practice.

**Design & setting:**

Exploratory qualitative study in Victoria, Australia.

**Method:**

Semi-structured interviews were undertaken to explore emotional health and support received in general practice in 12 adults with T2D who primarily attend general practice. Interviews were audio-recorded, transcribed verbatim, and analysed using the framework approach.

**Results:**

The following three major themes were identified: (1) Beneath the surface of diabetes care; (2) Importance of GP–patient relationship; and (3) Communication counts. Participants experienced diabetes care as focused primarily on medical management rather than the emotional aspects of living with T2D. While people’s experiences of diabetes care in general practice primarily focused on physical health, the GP care beyond the presenting complaint has an essential role in identifying emotional issues and enabling support. Emotional issues were more likely to be discussed and acknowledged by the GP where there was a long-standing relationship between GP and patient.

**Conclusion:**

Pre-existing positive GP–patient relationships and supportive communication enable people with 2TD to raise emotional issues as part of diabetes care.

## How this fit in

Despite guideline recommendations to assess for diabetes distress in people with T2D in general practice, this study found this was rarely experienced by people with T2D. General practice care was primarily focused on medical management but people with T2D also value general and diabetes-specific emotional support. Pre-existing positive GP–patient relationships and supportive communication enable people with T2D to raise emotional issues as part of diabetes care. People with diabetes value their GP acknowledging the invisible work of diabetes self-management, regardless of glycaemia.

## Introduction

T2D affects 90% of the estimated 537 million people worldwide living with diabetes. T2D impacts not only physical health but also emotional health.^1^ Diabetes distress describes the negative emotional reaction to living with and managing diabetes.^2^ Diabetes distress has been described for over 25 years in psychological literature, and up to 40% of people with T2D experience diabetes distress.^1^ Diabetes distress is linked to the daily experience of living with diabetes, and ongoing management. Elevated levels of diabetes distress are associated with increased risk of suboptimal glycaemia and self-management strategies; and has potentially more impact than depressive symptoms.^3–5^ Diabetes guidelines acknowledge the significance of emotional health in people with diabetes, with many recommending that healthcare professionals identify and address sources of diabetes distress.^6^ Suggested ways of identifying diabetes distress in clinical practice include using specific diabetes distress Patient Reported Outcome Measures (PROMs), such as the Problem Areas in Diabetes (PAID) scale. While these recommendations exist, studies reporting implementation of diabetes-specific measures in routine T2D in general practice are scare.^7^

Guideline recommendations to include emotional health assessment during diabetes care are consistent with the generalist model of care.^8^ However, several studies have indicated that these guidelines are not translating into practice. The second Diabetes Attitudes, Wishes and Needs (DAWN2) study indicated that almost two-thirds of healthcare professionals discuss emotional issues only if that discussion is initiated by the person with diabetes.^9^ International literature reports that people with diabetes do not recall having their emotional health addressed by a healthcare professional during diabetes care.^10^ In contrast, studies from general practice and specialist diabetes centres suggest that people with diabetes want to discuss their emotional health.^11,12^ In a recent survey of Australian GPs, only one in four reported routinely asking about diabetes distress.^13^ Qualitative findings from the UK indicated that discussion of emotional health is not part of the routine in diabetes care.^14^

The perspectives of people with T2D in Australian general practice have not been explored previously. This study aimed to explore the experiences, needs, and expectations of people with T2D regarding emotional health in Australian general practice.

## Method

### Study design

Semi-structured interviews were conducted between January 2021 and March 2021 with people with T2D who primarily attend general practice for diabetes care. This study is part of an exploratory multiphase research project, as part of the lead author's (RM's) PhD. The pragmatic paradigm was applied to this research. The pragmatic worldview focus is on the consequences of the research, the importance is on the questions asked, and on the use of multiple methods of data collection.^15^ Pragmatism employs a ‘what works’ approach using diverse approaches, including objective and subjective knowledge.^15^ The pragmatic paradigm it fits with is the lead author's worldview related to her work as a GP.

### Recruitment

A convenience sample of people with T2D were recruited using the Diabetes Victoria and the Australian Centre for Behavioural Research in Diabetes newsletter and websites, Diabetes Victoria private Facebook groups, the corresponding author's Twitter account, and the University of Melbourne staff newsletter. This included an advertisement to the study and contact details of RM. No people with T2D were approached directly for this study. Interested participants emailed RM and were provided with the explanatory statement and consent forms. A telephone or Zoom interview was then arranged, according to the participant's preference. People with T2D were eligible to participate if they primarily received T2D care in general practice. Participants received a $50 gift card and were welcome to share information about the study with eligible participants for snowball recruitment.

### Research team and reflexivity

RM conducted the interviews, and there were no non-participants present during the interviews. RM is a female specialist GP and a PhD candidate. RM was guided in the research by the co-authors JMN, BH, NFH, and JE. KS provided research assistant support. Researcher reflexivity involved attention to RM's and JMN's roles as specialist GPs providing care for people with T2D and was explored during weekly supervisory meetings with RM, JMN, and BH. None of the co-authors had a prior relationship with the study participants. Participants were made aware of RM's role as a specialist GP and PhD candidate in the explanatory statement before consenting and participating.

### Data collection

An interview guide was used (Supplementary File 1). The interview guide included questions about the general care provided by their GP, the participants’ experiences of emotional issues related to diabetes, their experience of emotional support offered in general practice, and their needs around emotional support. The guide also included questions about their experience of completing questionnaires to identify areas of concern about living with diabetes. After two initial interviews, the questions in the interview guide were reviewed and refined by RM and BH. Interviews were conducted remotely owing to the COVID-19 pandemic, using Zoom video technology or via telephone depending on participant preference, and were audio-recorded. No interviews were repeated, and no participants withdrew consent. RM kept a research journal to take reflective field notes after each interview. A professional medical transcription company was used to transcribe the data. Electronic copies of interview transcripts were shared with participants who had indicated in the consent form that they wished to review them.

### Data analysis

Data were analysed using a framework approach.^16^ Data collection and analysis occurred concurrently. The analysis consisted of the following six stages: familiarisation; identification of descriptive categories; indexing; charting; investigation; and interpretation. Data were managed using NVivo (version 12). RM checked the integrity of each transcript against the audio-recording. RM undertook familiarisation of the data through line-by-line reading of each transcript. RM coded the first three transcripts using an inductive and deductive approach (using the topic guide) to develop an initial coding framework. Three interviews were coded by KS, a qualitative researcher. Discrepancies between coding were discussed and the coding framework refined. The refined coding framework was then applied to the entire dataset by RM. Following coding of the entire dataset by RM, a framework matrix was developed using the refined coding framework. Key points identified were charted onto the framework with indexing to the original transcripts. The framework matrix was discussed with the other authors, and the codes were refined based on this discussion and structured into themes and sub-themes. Data are reported as per the COnsolidated criteria for REporting Qualitative research (COREQ) checklist.

## Results

### Participants

Seven women and five men participated in the interviews; age range 27–79 years (Table 1). The interview duration range was between 27 and 42 minutes. No participants withdrew from the study.

**Table 1.** Participant demographics

Interview number	Sex	Age, years	Years living with type 2 diabetes	Current glycaemic management
1	Male	70	21	Oral medication and insulin
2^a^	Female	53	1	Lifestyle
3	Female	60	3	Medication
4	Female	56	15	Oral medication and injectable GLP-1 agonist
5	Female	56	20	Oral medication
6	Female	73	30	Oral medication
7	Male	27	10	Oral medication and injectable GLP-1 agonist
8	Female	71	4	Oral medication
9	Male	74	20	Oral medication
10	Male	79	28	Oral medication
11	Male	70	0.5	Oral medication
12^a^	Female	40	2	Lifestyle

^a^Before diagnosis with type 2 diabetes, participant 2 and 12 had a diagnosis of gestational diabetes (17 years and 5 years ago, respectively). GLP-1 = glucagon-like peptide 1

### Themes

**Figure 1.**
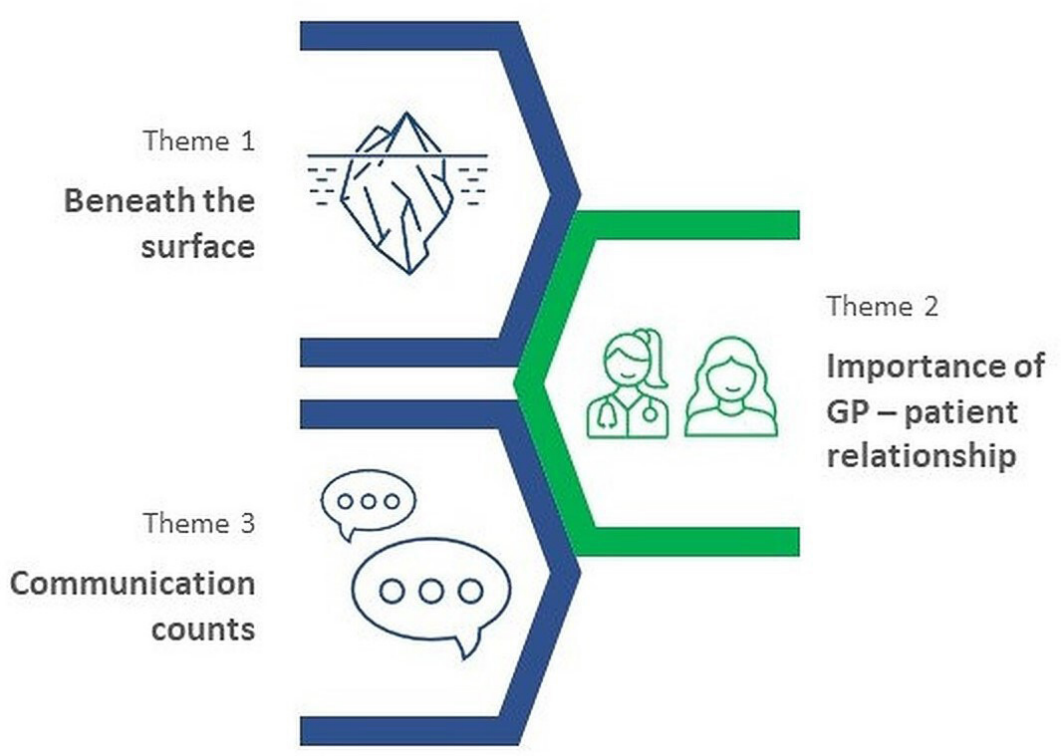
Theme map

The following three major themes were identified: (1) Beneath the surface of diabetes care; (2) Importance of GP–patient relationship; and (3) Communication counts. Although the themes are interlinked (see Figure 1), they are discussed individually.

#### Theme 1: Beneath the surface of diabetes care

Participants identified that their experiences of T2D were not confined to the diagnosis of the clinical condition but instead included emotional and wellbeing experiences hidden beneath the surface of diabetes care. Issues raised can be categorised into three sub-themes: shame; invisible work; and glycaemia and emotions. These were each described as contributing to the self-management and emotional health related to living with T2D.

##### Shame

Participants indicated that a diagnosis of T2D was associated with the feeling of shame, guilt, and failure. These feelings were expressed when considering disclosing a diagnosis of T2D, particularly owing to the negative social stigma related to T2D and weight. Some participants only disclosed a diagnosis to close family or a partner, with most expressing this person as an important support. One participant described guilt related to dietary measures resulting in increased comfort eating and subsequent guilt when visiting the GP:

*‘I know that if I make a decision to eat something that I know I shouldn't be eating or I don't control how much of what I'm eating. So, I think you can have a little bit of chocolate but then you keep going back and eating more and more of it. Yes. Then I feel guilty. Then I go to the doctors and I'm already feeling guilty before I even get there.’* (Participant 2)

##### Invisible work

Many participants described the cognitive load related to diabetes self-management, particularly related to dietary choices. This included the need to ‘give-up’ preferred foods and being vigilant when making dietary choices. While some participants described feelings of resentment related to lifestyle changes and fear of future complications, other participants developed positive self-talk related to their dietary choices and were more hopeful for the future:

*‘I think I’ve just developed my own inner strength with dealing with the whole thing and it’s just like, it’s okay, you’ll be okay, you’re doing the right thing, you can’t be too paranoid about what might happen down the track but if you do the right things now then hopefully, you’ll be fine.’* (Participant 12)

##### Glycaemia and emotions

Most participants described diabetes care as organised around testing glycaemia (using HbA1c) by the GP, with consultations to discuss the results. Participants reported that, at times, the emphasis was focused primarily on blood results and less on the person themselves. Several participants described that if HbA1c was at target, diabetes care tended to be primarily the delivery of blood results without further exploring current challenges in diabetes self-management. However, if HbA1c was suboptimal, care focused on lifestyle changes, particularly diet and weight loss, without examining their emotional health. Many participants expressed a desire to discuss further how emotions impacted glycaemia with their GP. Participants didn’t have the opportunity to raise the issues experienced in sub-themes 1 and 2 (shame and invisible work), which meant emotional issues went unaddressed:

*‘I think most of the conversation falls around basically … do you go for exercise, what’s your dietary requirements looking like? It hasn't really crept into the discussion around emotional distress and anxiety, but I think that’s probably something that I'd like to explore a little bit more. I think that’s obviously something that plays a part as well in terms of the highs and the lows of A1c.’* (Participant 7)

Participants who had diabetes for a longer period noted their levels of distress related to the diagnosis decreased with time:

*‘Initially as I said the family was a lot younger, all the kids were at home and that sort of thing. It rocked me a fair bit, but then when I found that it’s not — I can't say that it’s not a life- changing piece of information, but it’s not as dramatic as it might seem. It’s manageable, it’s easy to cope with. It’s not a bombshell.’* (Participant 9)

### Theme 2: Importance of GP–patient relationship

The quality of the relationship between GP and patient was identified as a core theme. Specifically, the length and stability of the relationship, the general physical and emotional support provided, and the respect that is given to the person with T2D influenced the discussion of emotional health.

#### Continuity of GP–patient relationship

Participants' experiences tended to differ based on their ongoing GP–patient relationship. Participants reflected that length of relationship with their GP, continuity of care, and previous positive experiences were facilitators of emotional support and communication. These benefits were aided by the GP also understanding the social and family context:

*‘Well, she’s probably known me for about maybe four or five years. So, she — and I pretty much only see her. So, she knows what’s been going on in my life. She also sees several members of my family, so she knows our family set up.’* (Participant 4*)*

Conversely, participants who had a more ‘matter of fact’ relationship with their GP did not think their GP was aware of their emotional concerns or could raise emotional issues:

*‘I think possibly for me, I'm pretty much a private and emotional person, so anything that I think might actually raise some emotions, I just try to redirect a question, do you know what I mean, I just really don't have that relationship with my doctor, and probably never have in fact with any doctor.’* (Participant 3)

#### GP understands the whole person

The GP’s ability to provide general emotional support, not just related to diabetes, contributes to the person with T2D’s perception of emotional support in diabetes care. While many participants had other healthcare professionals involved in diabetes care (including diabetes educators, dietitians, and endocrinologists), no participants indicated they received emotional support from these healthcare professionals. One participant indicated they attended a psychologist for a mental health diagnosis. Participants noted the importance of their GP understanding both their physical and emotional health:

*‘I think your GP needs to know you thoroughly and that includes your mental health as well as anything that’s fixable with meds or braces or whatever. I think your mental health is part of your overall health. It’s a holistic approach. A more rounded approach because your mental health affects how well you do physiologically, is my feeling. If you have a negative viewpoint, then you're going to take longer to recover from something, anything. So, I think your GP should be aware of that kind of thing. Whether you're feeling negative about things or not.’* (Participant 8*)*

Participants considered the GP to support them through the journey of living with T2D:

*‘I think from the GP my perspective would be mainly firstly to keep us motivated because obviously this disease, as we all know, is a life journey.’* (Participant 7)

#### Freedom to raise concerns

Finding and keeping a relationship with a GP was an essential aspect of emotional care in general practice. Participants expressed that their GP could understand their emotional needs because of the historical relationship and previous helpful responses of their GPs. Those reporting positive experiences tended to have lived with diabetes for a time (except one participant whose GP had recently retired). Having these foundations of a positive relationship allowed many participants to express emotional concerns with their GP:

*‘It’s* [emotional health] *never been a topic of conversation. I can easily turn to him and say, “right now I'm not coping with it”, and he certainly can come back and say “why not, what’s the story? What’s happening? What has happened?” I know that I could talk it through with him.’* (Participant 9)

Many participants noted that even if clinical consultations with their GP were short, their GP understood them as a person. The GP made time for general questioning beyond their presenting complaint during consultations. However, participants who experienced rushed consultations did not receive the required care. Some participants noted that their GP had limited time in consultations. Therefore, the person with T2D limited the emotional issues they shared with their GP, focusing more on biomedical information:

*‘It’s a time factor. I know she’s restricted with how much time she has for me. So, I keep my information limited so that I'm not holding her up, when maybe I actually need more time to be able to talk about the feelings as well.’* (Participant 2)

### Theme 3: Communication counts

The nature of communication, including strategies for gathering and delivering information, further impacts patient wellbeing. Key subthemes arising were the emotional impact of communication style, acknowledgement of effort required during diabetes self-management, and strategies to capture, filter, and discuss emotional wellbeing.

#### Communication impacts emotions

GPs’ communication styles impacted the participants’ emotional health, both positively and negatively. Factors contributing to positive communication included general questions about their personal life, including family, and using shared decision making when discussing management options:

*‘He cajoles you out of it, you know? If the question doesn't work that way, he'll put it around another way. I can't think of anything specific. Nothing’s coming to mind. I just know that I get more than my 15 minutes, and I 9.9 times walk out of his office feeling a hell of a lot better than I walked in.’* (Participant 9)

While shared decisionmaking was a strategy used by some GPs, other participants had the experience of their concerns not being heard by their GP, which negatively impacted their emotional health and even their focus on diabetes self-management:

*‘I feel now that I don’t have the support of the GP really so why should I bother, you know, worrying about my diabetes if he doesn’t seem to think it’s that important.’* (Participant 6)

#### Acknowledgement is needed

Many participants noted that more acknowledgement, praise, and encouragement related to the invisible work of diabetes self-management was needed to support their ongoing management and emotional health:

*‘Well, I think I would personally look to the HbA1c as the gold standard and that’s the thing that I aim to keep — that’s the parameter that I aim to keep within a good healthy range. So when you hit that, you just want somebody to give you a high five so — and not just go, well, there’s the bad side, there’s the bad bit here. Your fasting blood was a little bit high. So it was just a little bit disappointing, that’s all.’* (Participant 4*)*

Participants who had their self-management efforts acknowledged found it helpful in their ongoing self-management:

*‘The best thing that happened was, I went — actually I got one result from the one doctor when I went in. It wasn’t my normal doctor, and I got a blood — one of my blood sugars and she was like, “these are the best results I’ve ever seen for a Type two diabetic.” She’s like, “you’re smashing it, that’s awesome, keep doing what you’re doing”, and I was like, “awesome”. It’s just the reinforcement I get for having good results.’* (Participant 12)

#### Perception of a Patient-Reported Outcome Measure

Few participants recalled completing a PROM assessing the emotional aspects of living with diabetes, such as the PAID scale. Owing to previous experience with PROMs, as part of mental health care plans, some participants recognised the limitations of PROMs as a snapshot. One participant believed their GP knew them so well that their GP would already know how to respond to questions in a PROM. Conversely, participants who did not usually share their emotional concerns with their GP considered that using a PROM may increase their own and their GP’s awareness of unidentified emotional issues. Participants postulated that the GP having information about a person’s emotional health related to diabetes in writing may prompt GPs to pay more attention to emotional health:

*‘Because maybe it’s my GP, the lady that I see, who is lovely, but might not want to — might not know how to open up these conversations. Can look at that* [PROM responses] *and go, “oh, tell me about what you mean with this response” and then that opens up the dialogue where I can say, “well actually, I feel like this and this is embarrassing if I do this and have to tell the story to 1000 people, it’s boring, I’m sick of it” and it, yeah, maybe something like that would be good to get help the flow of conversation.’* (Participant 12)

## Discussion

### Summary

The main finding of this small qualitative study identified that, while people’s experiences of diabetes care in general practice primarily focused on physical health, the GP care beyond the presenting complaint has an essential role in identifying emotional issues and enabling support. Where issues exist beneath the surface, how the GP communicates with the person with T2D contributes to the emotional care received by people with T2D, including raising, acknowledging, and addressing those issues. The GP–patient relationship can facilitate discussing emotional issues, indicating that factors such as continuity of care may be significant for this patient group. Regardless of pathology results, people with diabetes value being asked about their emotional health and acknowledgement of its effects on diabetes self-management.

### Strengths and limitations

Strengths of this study are a focus on people with T2D who receive care in general practice, as literature in this area tends to include a sample of people with type 1 and T2D without a focus on general practice. Potential limitations include the exploratory nature, the use of a convenience sample of 12 people, and that people with T2D may have been more likely to participate if they had either positive or negative experiences in general practice. Diabetes distress is more likely in females, and in the present study female participants expressed more distress and shame related to their diagnosis.^3^ Research has indicated traditional masculine norms impact how men experience and express depressive symptoms, which may limit the findings of this study.^17^ The views and experiences of GPs will be explored in the next phase of research. Data collection for this study was undertaken in early 2021 in Victoria, Australia. The COVID-19 pandemic had impacted the delivery of care with an increase in telehealth consultations, which may have impacted experiences of diabetes care. The lead researcher is a specialist GP, which is a potential source of bias for the study. The ideas and assumptions of the researcher may impact the subjective nature of data analysis. In order to mitigate this, the multidisciplinary research team included non-GP researchers with whom data and themes were discussed.

### Comparison with existing literature

The findings of this study are consistent with the generalist approach to care, focusing on the individual parts while prioritising the whole person, including seeking connection with the person beyond a medical condition.^8^ ^18^ Other studies have also demonstrated that continuity of care is important to people with multiple medical conditions, and contributes to person-centred care and patient satisfaction.^19–22^ As in this study, a positive GP–patient relationship has been previously demonstrated to support the discussion of psychological issues.^3,23^

Despite diabetes guidelines promoting a focus on person-centred care, the present study highlights the ongoing focus on glycaemia in practice. Other qualitative studies have reported that doctors focus on glycaemic targets, which can be a barrier for people with diabetes to engage in meaningful discussions with healthcare professionals.^24–26^ These studies also found that healthcare professionals' communication may cause people with diabetes to disengage from medical care.^25–27^ Barriers to addressing emotional issues in general practice include difficulties disclosing emotional issues owing to perceptions of lack of time to address these issues, embarrassment related to the emotional issues, or owing to the doctor's communication style not facilitating disclosure.^28,29^ Poor communication in consultations is linked to higher diabetes distress.^30^ Training primary care staff in person-centred consulting skills has positive impacts on the person with diabetes’ perception of communication with their GP, satisfaction with treatment, and wellbeing.^18^ Building models of care that focus on the person's perception of their diabetes care, as opposed to the healthcare professional's, may facilitate care centred on the person's needs and enable them to raise issues related to their emotional health and to identify diabetes distress.

Results are consistent with previous surveys reporting that the use of the PAID scale to identify diabetes distress in Australian general practice is low, and internationally that only one-third of people with diabetes recall being asked about their emotional health by a healthcare professional.^10,13^ Other studies also indicate that people with diabetes want to talk about their emotional health.^12^ While people in the interview study did not recall the GP formally assessing diabetes distress using a PROM, many participants expressed that their GP knew about their emotional health owing to their previous GP–patient interactions. Participants who did not have an ongoing GP–patient relationship considered that a PROM may enable both them and their GP to understand their emotional concerns related to living with diabetes. This is in keeping with findings from Greenhalgh *et al*’s 2018 realist review, which found PROMs supported communication by enabling dialogue of issues not otherwise raised and allowing people to self-reflect on their medical condition.^31^ Current Australia policy in the 2020–25 National Health Reform Agreement recommends PROM collection across all levels of the health systems to support person-centred care and a focus on outcomes that matter to people receiving care.^32^ To support future use of any PROM collection and use in the clinical consultation, there are patient, healthcare profession, and environmental barriers that need to be addressed.^33^ Systematic review evidence suggests that studies of collection and use of diabetes distress and depressive PROMs in diabetes care are scarce; PROM implementation in diabetes care thus requires further exploration.^7^

### Implications for research and practice

This study explored the experiences of people with T2D in Australian general practice, focusing on core skills of general practice of developing a person-centred consulting style, with emphasis on the acknowledgement of the significant self-care burden of living with T2D. This study found pre-existing positive GP–patient relationships and supportive communication enable people with T2D to raise emotional issues as part of diabetes care. Diabetes guidelines now recommend addressing emotional health during diabetes care; however, this is not facilitated by existing funding structures and may be further negatively impacted by compliance activities that have targeted GPs with high rates of co-claiming both physical and mental health items in a single general practice appointment.^34^ Additional funding for the time needed to explore emotional aspects of diabetes care must be addressed. While funding models ae developed, future research can focus on pragmatic implementation of diabetes-specific PROMs to support identification of emotional concerns related to living with diabetes within the constraints of current care models.

Communication skills and the patient–doctor relationship are part of the core domains of general practice in Australia.^35^ In order to provide emotional support in general practice to people with T2D, GPs can focus on building general emotional support and relationships with their patients. People with diabetes value their GP acknowledging the invisible work of diabetes self-management, regardless of glycaemia. In practice, paying careful attention to the language healthcare professionals use with people with diabetes in order to support their self-management efforts is likely to facilitate person-centred care.^36^

## Data

The dataset relied on in this article is available from the corresponding author on reasonable request.
